# State patients who committed violent crimes and were admitted to Weskoppies Hospital from 2005 to 2014: Profiles and trends

**DOI:** 10.4102/sajpsychiatry.v26.i0.1416

**Published:** 2020-11-02

**Authors:** Zukiswa T. Dewet, Carla Kotzé, Funeka Sokudela

**Affiliations:** 1 Weskoppies Psychiatric Hospital, Pretoria, South Africa; 2 Department of Psychiatry, School of Medicine, Faculty of Health Sciences, University of Pretoria, Pretoria, South Africa

**Keywords:** state patients, violent crimes, trends, Weskoppies Psychiatric Hospital, psychiatry treatment

## Abstract

**Background:**

Most of the available literature focus on characteristics of violent offenders and trends of crime patterns in the general population. The crime trends in the population of those who may be mentally ill have not been well described.

**Aim:**

To describe the profiles and trends of mentally ill persons who committed violent crimes prior to admission as state patients.

**Setting:**

The study was conducted at Weskoppies Psychiatric Hospital.

**Method:**

A retrospective record review of state patients admitted between 2005 and 2014 was conducted to describe demographic, clinical, forensic and victim profiles of state patients admitted from 2005 to 2014. Trends over time were also assessed.

**Results:**

Hundred and seventy state patient files were reviewed. The majority were males (91.43%), with a history of substance use (55.0%) %), previous psychiatric treatment (46.4%), and diagnosed with a psychotic disorder (82.1%). The 10 year trend showed that murder was the leading charge during 2006 and 2007. It was surpassed by sexual assault crimes as the most common charge after 2007, except for 2009 when murder was again the most common. From 2010 onward, sexual assault remained the most common offence leading to admission as a state patient.

**Conclusion:**

This study found changes over time in crime patterns of state patients who committed murder and sexual assault. State patients may have different criminal patterns than the general public. This together with the high rates of substance use and previous psychiatric treatment can be important focus areas for future research.

## Introduction

South Africa is currently viewed as one of the most violent countries in the world.^1^ The Institute for Security Studies has coursed out patterns in the general population of all crime categories in South Africa at national and provincial levels from 2006 to 2018. Results show that from 2012 onwards, there is an increase in various categories of violent crimes. Violent crimes include aggravated robbery, murder, attempted murder and hijacking.^2^ Perpetrators of such crimes in the general population are mostly described as young males.^3,4^

Moreover, there is a perception that those who are mentally ill are more likely to commit violent acts than those in the general population.^5^ A literature review looking at public stigma and mental illnesses found that there was a growing perception in the United States of America that those who are mentally ill were dangerous.^6^ However, general consensus in literature on the relationship between mental illness and violence seems to be that having a diagnosis of mental illness alone does not predict future violent behaviour.^7^ What research has demonstrated, on the contrary, is that violence committed by those who are mentally ill arises from multiple factors that interact in a complex way.^4,7,8^

Literature has mapped out some of the factors that are of importance in understanding the relationship between mental illness and violent or criminal behaviour.^4^ The list includes demographic, historical, clinical and contextual factors.^4^ Demographic factors involved are the male sex, a younger age and a complex relationship between socio-economic status and criminal and violent behaviour amongst individuals with mental illness.^4^ Cited historical factors include past violence, juvenile detention, physical abuse and substance use and a history of victimisation. Contextual factors related to mental illness are recent divorce, unemployment and victimisation.^4,7,8^ Another variable of importance is the category of psychiatric diagnosis associated with criminal and violent behaviour. Whilst comparing psychiatric diagnostic categories with each other and against the general population, some have been shown to have a higher association with violent offenses.^4,9^ Laajasalo et al. reported that those diagnosed with schizophrenia and personality disorders were, respectively, 8 and 10 times more likely to be violent offenders when compared with the general population.^9^ This association was in the context of co-morbidities such as substance use/dependence.^9,10^

Characteristics of mentally ill offenders are well documented in the literature. A study conducted by Munetz et al. showed that psychiatric patients were generally at a greater risk of being arrested than the rest of the population. The study further showed that common diagnostic categories of those arrested were schizophrenia with co-morbid substance use.^11^ In South Africa a study involving six different forensic psychiatric units examined the psychosocial context and profiles of women offenders referred for psychiatric evaluation. It did not look at changes over time, but found that over the 12-year period in review, murder was the most common index offence and that there were high rates of psychotic and mood-spectrum disorders present in their sample.^12^ Another study that examined the factors associated with recidivism at Sterkfontein Hospital, South Africa, found that substance use disorder, antisocial personality disorder and having assault as an index offence were associated with a higher risk of recidivism.^13^

These available South African studies only described profiles of offenders; however, data illustrating changes in trends of the demographic and forensic characteristics in those who are mentally ill are lacking. This study was considered to be an important first step to address this gap in the data about changes in trends in mentally ill offenders, especially in a developing country context. A motivation for this study was the reported increase in violent crimes in the general population. However, the mentally ill are such a select population that correlations between this study population and the general population were not a focus of this study. The aim of the study was to describe trends of violent crimes committed by mentally ill people prior and leading to referral to a tertiary psychiatric hospital as state patients. Individuals charged with violent offences who have been found unfit to stand trial and/or who are found not to be criminally responsible because of mental illness or intellectual disability can be referred for care, treatment and rehabilitation as a state patient. They are referred by the court in terms of section 77(6)(a) or 78(6) of the *Criminal Procedure Act* 4 of 2017.^14^ The study further described the demographic, clinical and forensic data of state patients admitted to Weskoppies Hospital from 2005 to 2014. It also assessed whether there were changes over time in profiles of offenders, types of crime committed and aspects of victim profiles in this forensic population. This study also serves to add to the already existing body of literature and also offers to add a more contemporary perspective of this topic.

## Methods

The study was conducted at Weskoppies Psychiatric Hospital in Gauteng province, South Africa. It was a retrospective, record-based survey of state patients admitted to the Weskoppies Forensic Unit between 01 January 2005 and 31 December 2014.

A descriptive analysis was done looking at violent crimes committed by state patients and set out to assess trends over time. A total of 140 records of the total 170 state patients admitted over the study period were sampled. The rest of the files were missing. Included in the study were male and female state patients, older than 18 years of age who had committed a violent crime. A violent crime was described as one when the accused was charged with murder or culpable homicide or rape or compelled rape as provided for in sections 3 or 4 of the *Criminal Law (Sexual Offences and Related Matters) Amendment Act*, 2007, or another charge involving serious violence.

A data capturing sheet was used by the primary researcher to collect relevant data from all the selected files.

The three categories of variables included in this study were socio-demographics profiles, clinical profiles and variables related to the offence and victim profiles. Statistical analysis involved descriptive analysis by year or time categories, using frequencies and percentages. Comparisons were performed according to the year of admission in terms of demographics, patterns in crimes, state patient and victim profiles.

### Ethical consideration

Permission to conduct the study and review the relevant patient files was obtained from the chief executive officer of Weskoppies Psychiatric Hospital on behalf of Gauteng Department of Health as per regulations at that stage. Ethical approval was granted by the Faculty of Health Sciences’ Research Ethics Committee at the University of Pretoria (Ethical clearance number: 160/2017). All data were anonymised.

## Results

The clinical files of 140 patients, who were referred according to the *Criminal Procedure Act* to be admitted as state patients, were included in the study.

### Demographic characteristics

The age of individuals at admission ranged from 18 to 70 years, with a mean age of 33.4 (median of 31) years and standard deviation (s.d.) of 9.4 years. The demographic characteristics are summarised in Table 1.

**TABLE 1:** Demographic characteristics of study population.

Variable	Number	%
Gender		
Males	128	91.4
Females	12	8.6
Relationship status		
Single	103	73.6
Married	7	5.9
Divorced or separated	11	7.9
Widowed	3	2.1
Unknown	16	11.4
Employment status		
Employed	44	31.4
Unemployed	65	46.4
Pensioner	1	0.7
Unknown	5	3.8
Disability grant	25	17.9
Level of education		
None	7	5.0
Grades 1–7	35	25.0
Grades 8–11	50	35.7
Grade 12	22	15.7
Tertiary	6	4.3
Unknown	20	14.3

### Clinical characteristics

In the non-psychiatric diagnostic category, 47.9% of the study had no medical co-morbidities and 6.4% were unknown as they were not recorded in the patient files. In terms of psychiatric diagnoses, psychotic disorders (82.1%) were the leading diagnostic category for admission and prior to admission substances were used by 55.0% of those in our study population. The remaining 34.3% (*N* = 46) denied using substances prior to admission and 12.1% (*N* = 17) were not known as they were not recorded in the patient files. Almost half (46.4%) of the study population received psychiatric treatment prior to admission. Table 2 presents a summary of the clinical data.

**TABLE 2:** Clinical characteristics of study population.

Variables	Numbers	%
Medical co-morbidities		
Yes	64	45.7
No	67	47.9
Unknown	9	6.4
Medical co-morbidities		
HIV	19	29.7
Epilepsy	17	26.6
Head injury	4	6.3
Infectious other than HIV	2	3.1
Vascular (hypertension, diabetes mellitus)	20	31.3
Not specified	2	3.1
Psychiatric diagnosis		
Psychotic disorders	115	82.1
Bipolar and related disorders	1	0.7
Depressive disorders	3	2.1
Intellectual disability	13	9.3
Neurocognitive disorders	4	2.9
Unknown	4	2.9
Co-morbid substance use prior to admission		
Yes	77	55.0
No	46	32.9
Unknown	17	12.1
Previous psychiatric treatment prior to admission		
Yes	65	46.6
No	48	34.3
**Unknown**	**27**	**19.3**

HIV, human immunodeficiency virus.

### Yearly trends

**FIGURE 1:**
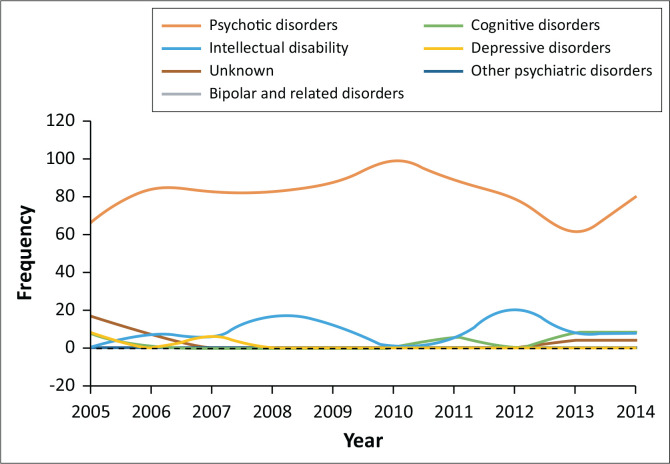
Frequency of different psychiatric diagnoses over the study period.

Throughout the 10-year period, those diagnosed with psychotic disorders showed the largest proportion of those admitted (see Figure 1). The trend in the admission patterns amongst those diagnosed with psychotic disorders oscillated throughout the 10-year period. In 2010 there were highest number of people admitted with psychotic disorders. This was followed by a slight downward trend in the psychotic disorder admission numbers, whilst there were slightly more admissions of individuals with intellectual disability and neurocognitive disorders after 2010.

**FIGURE 2:**
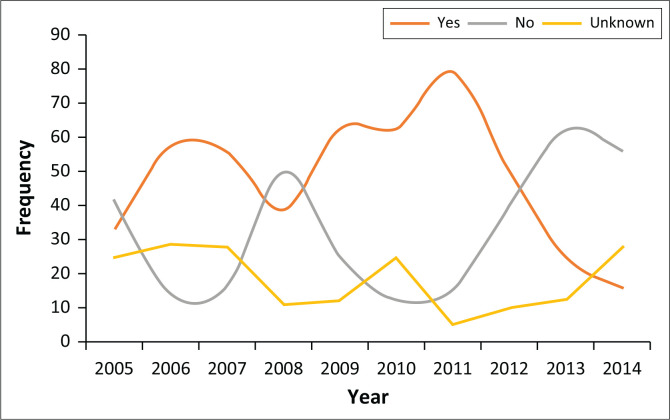
Frequency of previous psychiatric treatment over study period.

Those who previously received psychiatric treatment prior to admission as state patients were in the majority throughout the 10-year period except in 2008 and after 2011. This peak in 2011 was followed by a downward trajectory and was also noted to coincide with an increase in those who reported no previous psychiatric treatment use prior to admission and those who were noted to be unknown (see Figure 2).

**FIGURE 3:**
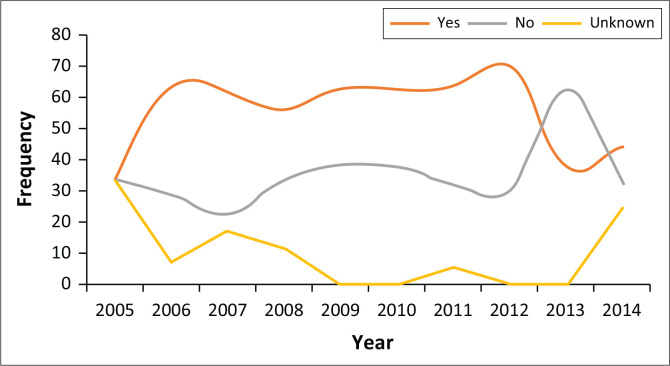
Frequency of history of substance use over study period.

The results in Figure 3 show that throughout the study period, there was a greater proportion of those who were admitted with a history of substance use until 2012 where a downward trend was noted. This downward projection coincided with an increase in the number of those who reported no previous history of substance use, with a peak in 2013.

### Forensic characteristics

Prior to the current admission, 45.7% of the population had previous arrests, 47.9% had no arrests and for 6.4% it was unknown. Those who were convicted of a crime prior to admission accounted for 15.7%, with 37.9% having no previous convictions and 46.4% being of unknown status. The majority of those admitted in the study period were charged with sexual offences (39.3%), murder (27.9%) and attempted murder (10.7%).

The forensic data including weapons used in the commission of the crime and the relationship of the victim with the state patient are presented in Table 3.

**TABLE 3:** Forensic data.

Variables	Number	%
Arrests prior to admission as state patient		
Yes	64	45.7
No	67	47.9
Unknown	9	6.4
Conviction prior to arrest leading to state patient admission		
Yes	22	15.7
No	53	37.9
Unknown	65	46.4
Current offence		
Murder	39	27.9
Attempted murder	15	10.7
Sexual assault crimes	55	39.3
Assault with intent to inflict grievous bodily harm/common assault	14	10.0
Robbery with aggravating circumstances	7	5
Other (arson)	10	7.1
Weapon of choice		
Firearm	12	8.6
Knives/cutting instrument	20	14.3
Bodily weapons (hands, fists)	54	38.6
Blunt objects	6	4.3
Strangulation/asphyxiation	2	1.4
Unknown	32	22.9
Other	5	3.6
No weapon used	9	6.4
Relationship of victim with perpetrator		
Child	8	5.7
Parent	7	5.0
Marital spouse/partner	8	5.7
Sibling	3	2.1
Other relative (aunt/uncle/grandparent)	10	14
Stranger	12	8.6
Unknown	51	36.4
Neighbour	32	22.9
No victim involved	5	3.6

**FIGURE 4:**
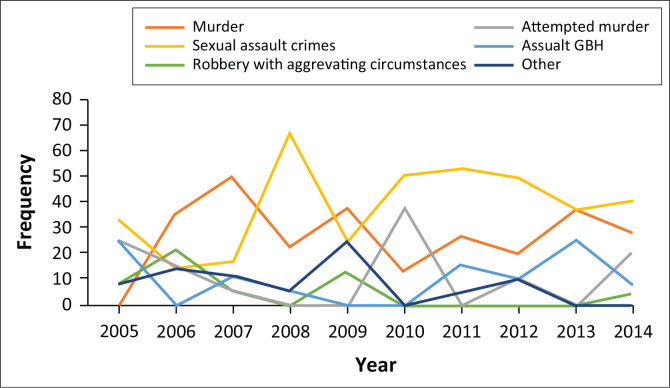
Frequency of different offences leading to admission over study period.

Figure 4 shows that those who committed sexual assault and murders were represented by a larger proportion of numbers throughout the 10-year period. The 10-year trend showed that the crime of murder was the leading charge at admission between 2006 and 2007. It was surpassed by sexual assault crimes when it became the most common charge at admission after 2007, except for 2009 when murder was again the most common offence for a 1-year period. From 2010 onwards, sexual assault remained the most common offence leading to admission as a state patient.

## Discussion

Psychiatric disorders have a notable prevalence amongst prisoners and other offenders.^15^ Forensic psychiatric units are regularly called upon to assess, treat, monitor and rehabilitate such offenders. Violent crimes are the usual reason for admission to forensic psychiatric units; however, one South African study found that there were cases of individuals being declared state patients even when they did not commit serious crimes.^16^ Notably from a South African perspective, there have been two studies from two academic centres that have looked at the profiles of state patients in a specific time frame.^17,18^ These two studies, however, did not investigate trends in their state patient profiles over a specific time. As a result of this, in our study we sought to describe the profiles and trends of mentally ill persons who committed violent crimes prior and leading to referral to Weskoppies Hospital as state patients. Throughout the 10-year period of the study, the demographic profiles of the state patients were generally in line with that found in local and international literature where the majority were male, single and unemployed.^16,17,19,20^

Psychotic disorders were measured as a broad category and constituted 82% of psychiatric diagnoses in our study, and it remained consistently high throughout the study period. The dominance of psychotic disorders amongst perpetrators of violent crimes is a well-reported phenomenon, but the number we found is higher than the norm.^17,18,19,20^ The higher numbers could be because of the fact that our study made no distinction between the different types of psychotic disorders and measured it as a broad category. Marais et al. found that 69% of their patients had psychotic disorders.^17^ Similarly, Barrett et al. found that 69% of their patients had psychosis and schizophrenia was diagnosed in 55.5% of the participants.^18^ In a study from South India, schizophrenia and other psychotic disorders made up 28.9% of diagnoses.^20^ Ibishi et al. reported that 69.1% of mentally ill offenders had a psychotic disorder,^21^ whilst Fioretti et al. found a prevalence of 65.6% for schizophrenia.^19^ These studies and our study support the notion that those who are likely to commit violent crimes within the psychiatric population are those diagnosed with psychotic disorders.^9,10^ The association between psychotic disorders and violent crime is often in the context of co-morbidities such as substance use and dependence.^9,10^ The proportion of co-morbid substance use in those diagnosed with psychotic disorders was not specifically investigated in our study.

Substances use is a general problem in society and our study also reflected this as a stand-alone phenomenon in our sample.^22^ Marais and Subramaney found that 59% of their sample in Johannesburg had a past psychiatric history and 71% had a substance use history.^17^ This is higher than that reported in our study where it was found that 47% had a past psychiatric history and 55% had a history of previous substance use. This may be a representation of the crime statistics in different cities within the province of Gauteng. According to the local crime statistics reported by the Institute for Security Studies, the city of Johannesburg had more drug-related crimes in comparison with Pretoria within the period 2006–2018.^2^ Generally substance use rates are much higher in psychiatric patients as compared with the general population and our findings further support this.^22^ Substance use in the context of a psychiatric diagnosis increases the risk of violent and criminal behaviour, which may partly explain the finding of higher rates of substance abuse in the forensic populations.^4,7,8^ The high rate of substance use has been a consistent finding in other South African studies and has been associated with longer admission periods and higher rates of recidivism.^13,23^

Individuals with personality disorders with co-morbid substance abuse were not evaluated in our study. This patient population generally accounts for a greater portion of those who commit violent crime in mentally ill offenders.^24^ Valenca et al. also found that those who present a mental disorder in combination with antisocial personality disorder and substance abuse/dependence are frequently refused treatment and commonly end up in the criminal justice system than by the mental health system.^24^ In our study, the absence of personality disorders as one of the diagnostic categories was noted to be an unusual finding in this forensic population and not in line with international studies. This has been similar in other local studies and a possible reason for this could be that personality disorders are often not the primary diagnosis when admission to a South African forensic psychiatric unit is required. This low number might be because of a missed diagnosis, poor documentation or it might be that the state patients have a different profile in South Africa.^13^ When it came to intellectual disability, it was the second most common diagnosis in our study population (9.3%). Although the low number of individuals with intellectual disability is different from international studies, it is in line with other studies conducted in South Africa on forensic populations.^13,18,25^

Almost half (46%) of our sample had previously been on psychiatric treatment, which implied that there was a pre-existing psychiatric diagnosis. A pre-existing psychiatric diagnosis was found in 59% of patients by Marais et al.^17^ Although our findings are in line with the literature, the slightly lower percentages in our study may be accounted for by the fact that 19.3% of participants were not known whether they had been on psychiatric treatment prior to admission or not. This is an important finding as previous psychiatric treatment and defaulting of treatment has a strong association with violent offending.^26^ The low number reported to have received previous psychiatric treatment could possibly be explained by poor history taking and reporting on initial interview at admission or poor record-keeping. It may also be explained by the fact that more patients have had poor access to mental health services prior to admission despite being mentally ill as is often the case in many parts of the country.^27^

A previous study from the Free State province of South Africa found that sexual assaults were the reason for 45.8% of their forensic admissions, followed by murder at 19.8%.^18^ Marais and Subramaney on the other hand found that sexual assault crimes were responsible for 26% of their admissions, whilst murder accounted for only 13% of admissions.^17^ Although there are variations in these statistics, in this study and the studies performed at other psychiatric units, sexual assault crimes have frequently been found to be the most common reason for admission.^17,18,19^ When we looked at the 10-year trends of reasons for admission, we discovered that sexual assault crimes were the most common offence leading to admission as a state patient since 2010. The two previously mentioned South African studies collected data from before 2008, and a more recent study conducted in KwaZulu-Natal looked at admissions between 2013 and 2016. This newer study had similar findings and rape was found to be the most common offence. No direct comparisons could be done with these studies because of differences in the timelines.^28^ Further investigation is warranted in the forensic populations of other South African forensic psychiatry units to test whether similar trends were seen in the same time period.

### Study limitations

The retrospective record-based nature of this study was a limiting factor as there were a number of files that could not be located and in many of the files the quality of the information recorded was insufficient. The small sample size, the lack of a control group and conducting the study in only one geographical location will limit the generalisability of the findings of this study. The factors investigated in this study were limited by the information available in the clinical files and other factors that might have contributed to the crimes committed, such as housing, family supervision and family structure, which were not within the scope of this study. Considering the complexities of forensic psychiatry, there is still value in sharing these findings and it might be valuable to replicate this study in other forensic psychiatry units in South Africa.

## Conclusion

In South Africa, similar studies of profiles of forensic patients have been performed, but our study offered a wider 10-year period looking into the profiles and trends of state patients admitted in a different geographical location in the country. Most of the characteristics of the state patient population found in this study are in line with those found in local and international literature. The consistent finding of high rates of individuals with a history of previous psychiatry treatment and substance use highlights this as an important area of future research, where the focus could be on possible interventions to minimise violent behaviour in this population. An analysis of the trends in the different categories variables showed that the overall patterns varied over the 10-year period. As there were no studies to compare this with, interpretation and implications of these findings will require further investigation. Despite it being so, our research may serve to highlight emerging trends in the forensic population. This provides opportunity for further research into this topic. For future research, prospective, longitudinal studies on a national level are recommended to look at changes over time in the characteristics of this patient population and the crimes committed by them.
